# The Impact of the COVID-19 Pandemic on Mental Health and Quality of Life in COVID-19 Department Healthcare Workers in Central Greece

**DOI:** 10.3390/jpm13020250

**Published:** 2023-01-29

**Authors:** Vasileios Aslanidis, Vasiliki Tsolaki, Maria Eirini Papadonta, Theofilos Amanatidis, Kyriaki Parisi, Demosthenes Makris, Epaminondas Zakynthinos

**Affiliations:** Critical Care Department, University Hospital of Larissa, Faculty of Medicine, University of Thessaly, Mezourlo, 41110 Larissa, Greece

**Keywords:** quality of life, mental health, ICU, general wards, healthcare workers, COVID-19, stress, anxiety, fear

## Abstract

Background: The pandemic crisis created conditions of insecurity and threat and brought about changes in social contacts and everyday life. Frontline healthcare workers (HCW) were mostly affected. We aimed to evaluate the quality of life and negative emotions in COVID-19 HCW and searched for factors influencing the above. Methods: The present study was conducted among three different academic hospitals in central Greece (04/2020–03/2021). Demographics, attitude towards COVID-19, quality of life, depression, anxiety, stress (using the WHOQOL-BREF and DASS21 questionnaire) and the fear of COVID-19 were assessed. Factors affecting the reported quality of life were also assessed. Results: The study involved 170 HCW in COVID-19 dedicated departments. Moderate levels of quality of life (62.4%), satisfaction with social relations (42.4%), working environment (55.9%) and mental health (59.4%) were reported. Stress was present in 30.6% of HCW; 20.6% reported fear for COVID-19, depression (10.6%) and anxiety (8.2%). HCW in the tertiary hospital were more satisfied with social relations and working environment and had less anxiety. Personal Protective Equipment (PPE) availability affected the quality of life, satisfaction in the work environment and the presence of anxiety and stress. Feeling safe during work influenced social relations and fear of COVID-19 Conclusion: The HCW quality of life is affected in the pandemic. Feelings of safety during work related to the reported quality of life.

## 1. Introduction

The coronavirus disease 2019 (COVID-19) pandemic has brought health challenges worldwide, and many populations are expected to face adverse mental conditions [1]. Healthcare workers (HCW), who have been on the front line from the very first moment of the crisis, are considered high risk populations for such adverse conditions, not only due to the increased workload, but also due to the increased risk of direct exposure and the limited availability of Personal Protective Equipment (PPE) [2]. Compared to the general population, there is a twelve-fold increased risk in health care workers to test positive for COVID-19 [3]. 

Healthcare workers already face increased levels of work-related stress and exhaustion due to the demanding nature of the work and quality of care they are required to provide [2]. The additional load during the pandemic was associated with negative emotions such as depression, anxiety, insomnia, exhaustion and significant stress at work [4]. In addition, isolation measures, fear, uncertainty, economic instability, social disconnection and lack of trust in other people and institutions, became new psychological stressors [5,6,7,8,9]. Fear was a new, added emotional burden, concerning the fear of being infected and the fear of transmitting the virus to their families.

In addition, organisational factors (such as changes in the working environment, the institutional climate, moral harassment, excessive workload, low wages, among others), but especially the severity of the pandemic, can cause emotional exhaustion [10,11]. In contrast to these situations, a trend that emerged was the accreditation to health professionals a pro-hero status. This, while adding value, also poses additional stress due to the increased demands involved, the media exposure and the shocking nature of an event of global proportions, increasing the need for emotional support, encouragement and appreciation [12].

The aim of the present study was to investigate the quality of life (QOL) and negative emotions in COVID-19 healthcare workers from the beginning of the pandemic (April 2020) until the end of the second wave (March 2021). In addition, we tried to identify factors affecting the quality of life and the presence of negative emotions. To our knowledge, this is the first study to evaluate the quality of life of healthcare personnel, working in dedicated COVID-19 departments in Greek hospitals and among different academic degree hospitals.

## 2. Materials and Methods

### 2.1. Study Design-Sample

The study was conducted in 3 different academic hospitals in Central Greece (University General Hospital of Larissa (UHL) (650 bed-capacity), General Hospital of Larissa (GHL) (220 bed-capacity) and General Hospital of Trikala (GHT) (190 bed-capacity)), from April 2020 to December 2021. The study was approved by the Local Ethics Committee (Νo 3375/2020). Participants’ anonymity, voluntary participation and the right to withdraw were confirmed. The completion of the questionnaire indicated their consent to participate in the survey. The necessary ethical considerations that are related to the nature of a research and to the psychology of participants were clarified [13]. The population of the survey is the total number of frontline healthcare workers in COVID-19 departments (Emergency Department, COVID-19 dedicated general wards, COVID-19 ICU) during the pandemic period in Thessaly, Central Greece. The study questionnaire was sent by e-mail to all frontline healthcare workers of the three participating hospitals. 

### 2.2. Survey Design

A primary, quantitative, non-experimental survey was conducted using Likert scale questionnaires (World Health Organization Quality of Life Assessment -WHOQOL-BREF and the Depression Anxiety and Stress Scale 21 (DASS21) questionnaires were used) (Appendices A and B) [14,15]. The dependent variables of the survey were the factors of quality of life, physical, mental health, satisfaction from social relationships and working environment, stress, anxiety, depression and fear of being infected by SARS-CoV-2. Independent variables of the survey were demographics, job characteristics and impact of social media. Quantitative research was considered appropriate as the factors of quality of life and negative emotions are measurable values and because it is necessary to investigate correlations between the variables in order to generalize the conclusions [16]. The non-experimental design lies in the fact that the research explores the factors associated with reduced levels of quality of life and negative emotions, without looking for a cause-and-effect relationship [17]. 

### 2.3. Survey Questionnaire

The distributed questionnaire consisted of 59 questions divided into 4 sections. The 1st section referred to the demographic and professional experience data, using 7 questions. The 2nd section included 10 questions about attitudes concerning the pandemic. The 3rd section included 18 questions on quality of life, according to the World Health Organization Quality of Life Assessment -WHOQOL-BREF questionnaire [14]. The 4th section measures the negative emotional states of depression, anxiety and stress using the Depression Anxiety and Stress Scale 21 (DASS21) questionnaire [15] and 6 questions from the Fear of COVID-19 Scale questionnaire [18,19], which have been validated in previous surveys and translated in the Greek language. The DASS21 questionnaire indicated acceptable reliability for stress (a = 0.603) and depression (a = 0.650) and satisfactory for anxiety (a = 0.706). The Fear of COVID-19 Scale showed high reliability (a = 0.806). Fear was evaluated with two general questions and five standardized questions using the Fear of COVID-19 Scale questionnaire. The questions are answered on a 5-point Likert scale from 1–5. Values between 1 and 2.60 were considered low, 2.61 and 3.40 were moderate and between 3.41 and 5 were high.

The study period covered the 1st pandemic wave, before the vaccine was launched, and the 2nd wave during which the first vaccine was available. The questionnaire was answered once by each participant.

### 2.4. Statistical Analysis

Scale variables were presented with mean and standard deviation, whereas nominal variables were presented with percentages and frequencies. The normality of variables was tested using the Shapiro–Wilk test. Normally distributed continuous variables were compared with independent sample t-test (between two groups), or ANOVA for multiple group comparisons; non-normally distributed variables were compared via the Kruskal–Wallis. Spearman correlations were performed between scale non-normal variables and Pearson correlation was used for continuous variables. Test reliability was performed using the Conbach alpha Reliability analysis of the statistical package. Predictors of dependent variables were identified using multiple linear regression analysis. Nominal variables were entered in the model using dichotomous variables (0 = No, 1 = Yes) for each category. Data were analyzed using SPSS (IBM SPSS statistics version 25). A *p*-value < 0.05 was considered statistically significant.

## 3. Results

### 3.1. Demographic Characteristics

During the study period, covering two epidemic waves, almost half of the hospital wards and the majority (80%) of ICU beds were dedicated to COVID-19 patients and the beds overwhelmed. The questionnaire was distributed in all healthcare workers of the three hospitals (230 HCW) and 170 questionnaires were returned (73.91%). All the questionnaires were fully answered (there were no missing data). The majority were females (64.7%, N = 110) and married (55.9% (N = 95)). In total, 106 HCW (62.4%) were working in the UHL and 64 (37.6%) in the GHL and GHT, whereas 52.9% (N = 90) were working in the intensive care units (ICUs), 29.4% (N = 50) in the emergency departments and 17.6% (N = 30) in the COVID-19-dedicated departments. The study sample included nurses (64.7%, N = 110) doctors (23.5%, N = 40) and other health professionals (ward assistants and workers in the cleaning services, (11.8%, N = 20)). Concerning the working experience, 61.8% (N = 105) had been working for less than 10 years (Table 1).

### 3.2. Infection-Vaccination Status

The majority of the responders reported that they had not been infected with COVID-19 (76.5%, N = 130), 75.9%, (N = 129) were waiting for the release of the vaccine (during the first wave) and 84.7% (N = 144) as health professionals, would recommend vaccination. However, 29.4% (N = 42) had not been vaccinated against coronavirus during the first three months of vaccine distribution. The vaccination was voluntary, during the research period, among healthcare workers.

### 3.3. COVID-19 Information

Among social media, the internet was the most common source of information during the pandemic period (67.1%, N = 114), the majority reported feeling scared during the pandemic (67.1%, N = 114) and, among them, more fear was reported during the second pandemic wave (60.5%, N = 69). The general feeling for the impact of the mass media during the pandemic was considered negative by the majority (61.2%).

### 3.4. Quality of Life and Negative Emotions

General quality of life was moderate (62.4%); apart from high levels of physical health (73.5%), moderate levels were reported for the remaining domains. Regarding emotions, most healthcare workers reported low levels of stress (69.4%), fear of COVID-19 (79.4%), depression (89.4%) and anxiety (91.8%), using the standardized DASS21 and Fear of COVID-19 questionnaires; although they had reported generally feeling scared (67.1%), when a standardized questionnaire was used [19], it reported low levels of fear (Table 2 and Table 3).

Most of HCW reported that the working institution consistently provided the required PPEs (57.6%); however, only 16.5% felt safe during work tasks with infected patients and half of HCW felt unsafe (52.9%) (Table 3).

### 3.5. Factors Influencing Quality of Life and Negative Emotions

The results concerning the effect on quality of life in different subpopulations are presented in Table 4.

Gender and marital status: Males had better mental health than females (*p* = 0.019) but were less satisfied with their social relations (*p* = 0.005). Divorced/widowed HCW were less satisfied with their work environment and social relations compared to the unmarried and married (*p* < 0.001 and *p* = 0.002, respectively).

Working Institution: Satisfaction from the working environment was higher in HCW of UHL than GHL and GHT (*p* < 0.001). Similarly, satisfaction from the social relations was highest in UHL HCW (*p* = 0.007) and significantly differed between HCW of the two general hospitals (3.11 ± 0.82 (GHL) vs. 2.68 ± 0.55 (GHT), *p* = 0.040). Anxiety was significantly higher in HCW in the GHL compared to the UHL (*p* = 0.036).

Working Department: Satisfaction from working in an ICU environment was significantly higher than working in the general ward/emergency department (*p* < 0.001) and ICU HCW were more satisfied from their social relations than workers in other departments (*p* = 0.005).

Working Position: Doctors reported higher levels of quality of life in the various indices, compared to nurses and other HCW: mental health (*p* = 0.032), satisfaction with the environment (*p* = 0.023) social relations (*p* = 0.028). In addition, depression was significantly lower (*p* < 0.001), and doctors experienced less fear (*p* = 0.011).

Work experience: HCW with longer work experience were more satisfied with the work environment (*p* = 0.005) and social relations (*p* = 0.035).

Source of information: Quality of life was better among HCW relying their COVID-19 information on sources other than television and internet, and they reported increased satisfaction with the social relations (*p* = 0.002), less stress (*p* = 0.002) and depression (*p* = 0.046) (Table 4).

The effect of mass media: The subjective effect of mass media on personal life was positively associated with environmental working satisfaction (r = 0.286, *p* < 0.01) and social relations (r = 0.308, *p* < 0.01) while negatively associated with anxiety (r = −0.224, *p* < 0.01), meaning that people who perceived a negative impact had worse working satisfaction and social relations and higher anxiety.

### 3.6. Factors Affecting Quality of Life and Negative Emotions

Simple correlations between possible factors affecting the quality of life and negative emotions are presented in Table 5.

Multivariate regression analysis revealed that the quality of life was associated with PPE availability (b = 0.252, *p* = 0.002). Mental health was affected by gender (b = −0.168, *p* = 0.024) and PPEs (b = 0.218, *p* = 0.007). Satisfaction from the work environment was affected by safety during work (b = 0.290, *p* < 0.001), PPE availability (b = 0.233, *p* = 0.001), the marital status (b = −0.178, *p* = 0.007) and source of information (b = 0.139, *p* = 0.029), whereas satisfaction from social relations was associated with the use of mass media (b = 0.180, *p* = 0.020) and a safety feeling during work (b = 0.180, *p* = 0.021). Fear for COVID-19 was negatively associated with safety during work (b = −0.301, *p* < 0.001). Depression was associated with the profession (b = 0.382, *p* < 0.001) and the source of information (relying on TV b = 0.200, *p* = 0.006). Anxiety and stress were affected by PPE availability (b = −0.175, *p* = 0.033 and b = −0.198, *p* = 0.013, respectively). Finally, safety during work decreased the levels of anxiety (b = −0.169, *p* = 0.048) (Table 6).

## 4. Discussion

The present study investigated the quality of life and presence of negative emotions during the pandemic in COVID-19 healthcare workers in central Greece and showed that the quality of life was affected during the pandemic. The most vulnerable populations identified were women, singles and healthcare workers in secondary hospitals. The social media negatively affected the physical health, social relations and environmental work satisfaction, whereas television/internet enhanced levels of depression and stress.

In the present study, we have evaluated the quality of life during the pandemic in different groups of HCW according to their institution, department, profession and work experience. The overall QOL was rather good (moderate levels reported the 62.4% of HCW); physical health levels were not affected, whereas mental health was moderate in 59.4% of HCW. It seems that the QOL is better in Greek HCW compared with the reported values from other countries [20,21]. Shreffler et al., in a review of 37 studies covering the first pandemic wave, found that there were consistent reports of stress, anxiety and depressive symptoms in frontline workers, and 10–28% reported moderate to severe levels of depression and anxiety, as a result of the COVID-19 pandemic [22]. HCW in China experienced increased levels of distress (71.5%) and depression (50.4%), whereas anxiety (44.6%) and insomnia (34.0%) were lower. HCW (doctors and nurses) in Belgium, reported 28.8% depression, 25.1% distress, 36.8% insomnia, and 36.1% of the medical staff presented moderate to severe symptoms of anxiety [23,24]. The collective data showed that 88.3% of healthcare personnel worldwide, experienced at least mild levels of stress or emotional deviations [22]. On the contrary, our findings indicate lower stress levels among HCW (medium to high stress levels were reported in 30.4% of the participants, anxiety in 8.2% and depression in 10%), which is surprising, considering the period that the study covered, with the highest burden of COVID-19 hospitalized patients. Adequate PPE provision, by ensuring the feeling of safety, was associated with less fear and anxiety and better overall quality of life, and may explain these findings. Moreover, satisfaction from the work environment, possibly due to communication strategies, may have contributed.

This is the first study to evaluate the quality of life of frontline HCW in Greece. To our knowledge, only one study has evaluated mental health of HCW, and it concerned the post-traumatic stress and coping strategies of all health professionals, only during the first wave of the pandemic, without focusing on the quality of life of those working in COVID-19 departments and all the factors that affect the appearance of negative emotions [25]. Kalaitzaki et al., reported that 79.3% of HCW presented at least moderate Secondary Traumatic Stress, although stress was not evaluated in different subgroups of HCW [25]. Another Greek study in HCW, 27% of whom were working in the frontline, reported moderate levels of professional quality of life and occupational stress [26], whereas another study focusing on mental HCW reported low overall mean fear of COVID-19 [27]. In our study we found that negative emotions were not very common in HCW; medium to high levels of fear for COVID-19 were present in 21.6% of HCW, depression in 10.6%, anxiety in 8.2% and stress in 30.6%. Moreover, negative emotions varied according to the working institution, department, profession, and work experience. Higher levels of fear have been attributed to social disconnection and trust in other people [5,6], elements that seem to have been less pronounced in the currently studied population. The quality of communication and friendly relationships in the work environment affects the levels of stress [22]. Low levels of negative emotions among the study population may be attributed to the reported moderate to high levels of satisfaction with the work environment (87.1%) and social relations (82.4%).

### 4.1. Quality of Life Depending on Different Subgroup Categories

#### 4.1.1. Gender

Men had better mental health but were less satisfied from their work environment and social relations in the present study. Enestrom et al. attributed this difference to lower support from family or partner and decreased ability to express the need for support [28]. Negative emotions did not differ between males and females in the present study. Women in high-risk areas may present more negative psychological outcomes, as they have to deal with a greater workload, added to the burden of home responsibilities [20,22]. The marital status was another factor affecting the quality of life in the studied population. The presence of a partner to share the reality has been identified as a crucial factor for satisfaction with the environment and social relationships [28].

#### 4.1.2. Working Institution

The working institution and department had an additional impact on HCW quality of life. Healthcare workers in secondary hospitals were less satisfied with the work environment and social relations compared to the workers in the tertiary hospital. They reported less satisfaction with their social relationships and higher levels of anxiety. Our findings of better HCW quality of life in the tertiary hospital may be explained by the application of protocols and higher levels of interdisciplinary communication and updated information. In other institutions, highly qualified personnel on psychological issues may also have a positive impact [29]. In Greece, secondary hospital HCWs had to face a greater lack of resources, personnel and information compared with tertiary hospitals [30].

#### 4.1.3. Working Department

Intensive care unit HCW reported increased satisfaction with the environment and social relationships compared to workers in other departments. To our knowledge, this is the first study to report such a difference during the pandemic, although the rate of burn out in such environment is more frequent [31,32]. Before the pandemic, it had been recognized that ICU staff have better environment and social relations than workers in other departments, attributed to the higher level of specialization and the strict protocols that are followed [29].

#### 4.1.4. Profession

Doctors and nurses reported higher satisfaction with the environment and social relationships and lower levels of depression than other workers. This contrasts with the findings of other studies, which report that front-line hospital workers (doctors, nurses) reported the least job satisfaction, and the highest levels of burnout [31,33]. It is likely that dedication, teamwork and the acknowledgement of offering services to vulnerable populations may explain these findings. Nurses presented higher levels of depression and fear of illness, which could result from closer contact with patients and more intense experience of patients’ pain and loss [12].

#### 4.1.5. Working Experience

The length of work experience affected the satisfaction with the working environment; longer experience was associated with increased satisfaction, which is in accordance with the findings of Mihalache et al., reporting that satisfaction with the environment and social relationships is related to the working experience, level of specialization, job insecurity and organizational support [34]. Stefanatou et al., reporting similar results concerning the working experience, pointed that older age enhances the ability to derive satisfaction from the working environment and deal with adversities [27]. In the pandemic era, as relationships with organisations were in a delicate balance, HCW with more experience showed higher levels of satisfaction.

The majority of healthcare workers had not been infected by coronavirus but knew people in their work environment who had. Despite the adequacy of PPEs, 52.9% of the HCW reported feeling unsafe while working with COVID-19 patients. Lower feelings of safety and inadequate PPEs correlated with lower quality of life, and higher levels of fear of COVID-19, anxiety, stress and depression. Accordingly, the sense of security among HCW was limited during the pandemic and was counterbalanced by the provision of adequate PPEs [6,35]. Therefore, the adequacy of PPEs and mental support are key factors for healthcare worker’s physical and mental health, especially during crises [34].

Mass media posed a negative impact on quality of life (61.2%). Workers who relied on the internet and television as a source of information presented lower levels of physical health and satisfaction with the environment as well as higher levels of fear of illness and worry compared to workers who relied on other sources of information. People who perceived the negative impact of the mass media during the pandemic tended to experience worse physical health and less satisfaction from environment and social relations while reporting higher stress and anxiety. To our knowledge, the present study is the first to associate the negative impact of the social media on HCW’s mental issues and psychology. Health literacy has been shown to improve QoL during the COVID-19 pandemic [36]. The social media have been found to increase the feeling of stress and affect the mental health in the general population as well as university students during the pandemic [37,38].

Regarding attitudes about vaccination, most healthcare workers, during the first epidemic wave, reported that they were waiting for the release of the vaccine to eliminate the COVID-19 pandemic, have had a vaccination against coronavirus disease, when the vaccine was released, and would recommend vaccination. Globally, HCW had a positive attitude concerning the vaccine against COVID-19 [39]. Nevertheless, resistance to the vaccine among health workers was particularly high in Greece, with the first surveys at the beginning of the pandemic and before the vaccination campaign, showing only 51.1% positivity to receive the vaccine [40]. In our study, vaccination rate was high, although the period covered only the first three months from vaccine distribution, and this could be attributed to the nature of working conditions, as all HCW dealt with COVID-19 patients.

### 4.2. Limitations-Suggestions

The sample size was relatively small, yet it is rather representative of the healthcare workers in central Greece as it comprised frontline HCW from one tertiary and two secondary hospitals, severely affected from COVID-19. Moreover, the study population covers a rural area, excluding residents in large urban areas such as the capital of Greece, where other factors such as alienation, might have a further impact on quality of life. Another strength of the study is that it included HCW from different departments, with inherent differences in workload, demanding tasks and emotional involvement. Certainly, a nationwide survey is warranted to further explore the differences and possible factors affecting them, among HCW working in various academic degree positions, departments and with different workload. It should also be pointed that HCW quality of life and negative emotions were assessed once and there was no comparison between the two pandemic waves for each HCW. Moreover, all departments (EDs, wards and ICUs) were overwhelmed with COVID-19 cases during the study period.

## 5. Conclusions

In conclusion, the results of the present study indicate that the quality of life of frontline healthcare workers has been negatively affected by the pandemic. The most vulnerable populations identified were women, singles and healthcare workers in secondary hospitals. Surprisingly, HCW in the ICU presented higher quality of life compared to HCW in other dedicated COVID-19 departments. Teamwork and adherence to protocols in the working place may have affected the results and should be encouraged in every institution. Health literacy by updated accurate and transparent information is another goal to support HCW. Ensuring the sense of security in the working place, including the adequate provision of PPEs, is a necessity to improve quality of life and decrease the fear of COVID-19.

## Figures and Tables

**Table 1:** Demographic and occupational data.

Demographics	Categories	Ν = 170	%
Sex	Female	110	64.7
Age	Up to 30	27	15.9
	31–40	70	41.2
	41–50	63	37.1
	>50	10	5.9
Marital Status	Unmarried	50	29.4
	Married	95	55.9
	Divorced	24	14.1
	Widower	1	0.6
Working Institution	Tertiary	106	62.4
	Secondary	63	37.6
Department of Work	ICU	90	52.9
	Emergency Department *	50	29.4
	Internal Medicine Department *	5	2.9
	Respiratory Department	6	3.5
	Department of Infectious Diseases	12	7.1
	Other	7	4.1
Profession	Doctor	40	23.5
	Nurse	110	64.7
	Physiotherapist	6	3.5
	Paramedic	5	2.9
	Ward Assistant	5	2.9
	Cleaning services	4	2.4
Working Experience (Years)	Up to 5	53	31.2
	6–10	52	30.6
	11–15	31	18.2
	>15	34	20.0

ICU: Intensive Care Unit. * Internal Medicine Department and Respiratory Department were hospitalizing COVID-19 patients and were considered as subdivisions of the Department of Infectious Diseases.

**Table 2:** Quality of life and negative emotions.

Factor	M	S.D.	Low	Medium	High
Quality of life	2.87	0.76	24.1%	62.4%	13.5%
Physical health	3.58	0.58	4.7%	21.8%	73.5%
Mental health	3.11	0.62	17.6%	59.4%	23.0%
Work Environment	3.15	0.52	12.9%	55.9%	31.2%
Social relations	3.30	0.92	17.6%	42.4%	40.0%
Fear of COVID-19	2.07	0.69	79.4%	17.6%	3.0%
Stress	2.46	0.59	69.4%	22.4%	8.2%
Anxiety	1.73	0.59	91.8%	5.9%	2.3%
Depression	1.90	0.61	89.4%	6.5%	4.1%

The questions are answered on a 5-point Likert scale from 1 to 5. Values between 1 and 2.60 were considered low, 2.61 and 3.40 moderate and between 3.41 and 5 high.

**Table 3:** Impact of the pandemic on daily life.

Nominal Variable	Categories	Ν = 170	%
Infected *	Yes	40	23.5
Relative infected	Home environment	4	2.4
	Working environment	98	57.6
	Friendly Environment	15	8.8
	All the above	38	22.4
	Nobody	15	8.8
Source of information	Internet	114	67.1
	Television	37	21.8
	Other	19	11.2
Feeling of fear about the COVID-19 pandemic	Yes	114	67.1
Pandemic period with the highest fear feeling	First Period	45	39.5
	Second Period	69	60.5
Waiting for the release of the vaccine (before December 2020)	Yes	129	75.9
Vaccinated	No	42	29.4
Recommendation of vaccination to others	Yes	144	84.7
Impact of social media during COVID-19	Negative	104	61.2
	Neutral	46	27.1
	Positive	20	11.8
Feeling of safety during work with COVID-19 patients	Low	90	52.9
	Medium	52	30.6
	High	28	16.5
Adequacy of PPEs	Low	17	10.0
	Medium	55	32.4
	High	98	57.6

COVID-19: coronavirus disease 2019; PPE: Personal Protective Equipment; * during the whole study period.

**Table 4:** Comparisons between quality of life, fear of COVID-19 and negative emotion variables among different sub-populations.

	Mental Health	Physical Health	Quality Of Life	Work Environment	Social Relations	Fear of COVID-19	Depression	Anxiety	Stress
Gender [Male (60) vs. Female (110)]	3.26 ± 0.59 vs. 3.02 ± 0.62 *p* = 0.019	3.62 ± 0.48 vs. 3.56 ± 0.64 *p* = 0.499	2.97 ± 0.76 vs. 2.82 ± 0.76 *p* = 0.223	3.08 ± 0.46 vs. 3.19 ± 0.54 *p* = 0.170	3.06 ± 0.69 vs. 3.43 ± 1.00 *p* = 0.005	1.95 ± 0.57 vs. 2.13 ± 0.73 *p* = 0.098	1.99 ± 0.64 vs. 1.85 ± 0.60 *p* = 0.156	1.66 ± 0.41 vs. 1.77 ± 0.67 *p* = 0.218	2.41 ± 0.57 vs. 2.49 ± 0.60 *p* = 0.382
Marital Status [Unmarried (50) vs. Married (95) vs. Divorced-Windowed (25)]	2.99 ± 0.75 vs. 3.16 ± 0.58 vs. 3.15 ± 0.44 *p* = 0.269	3.58 ± 0.63 vs. 3.62 ± 0.60 vs. 3.45 ± 0.40 *p* = 0.447	2.92 ± 0.78 vs. 2.87 ± 0.80 vs. 2.76 ± 0.52 *p* = 0.705	3.27 ± 0.54 vs. 3.19 ± 0.50 vs. 2.77 ± 0.33 *p* < 0.001	3.25 ± 1.08 vs. 3.45 ± 0.85 vs. 2.85 ± 0.61, *p* = 0.007	2.05 ± 0.73 vs. 2.06 ± 0.71 vs. 2.14 ± 0.52 *p* = 0.846	1.87 ± 0.59 vs. 1.84 ± 0.55 vs. 2.22 ± 0.78 *p* = 0.091	1.74 ± 0.56 vs. 1.72 ± 0.67 vs. 1.75 ± 0.33, *p* = 0.949	2.44 ± 0.69 vs. 2.43 ± 0.57 vs. 2.63 ± 0.40 *p* = 0.301
Working Institution [UHL (106) vs. GHL (38) vs. GHT (25)]	3.06 ± 0.69 vs. 3.12 ± 0.52 vs. 3.31 ± 0.39, *p* = 0.172	3.66 ± 0.65 vs. 3.45 ± 0.46 vs. 3.42 ± 0.31, *p* = 0.076	2.87 ± 0.87 vs. 2.93 ± 0.53 vs. 2.79 ± 0.51 *p* = 0.723	3.28 ± 0.57 vs. 3.01 ± 0.35 vs. 2.85 ± 0.28 *p* < 0.001	3.51 ± 0.94 vs. 3.11 ± 0.82 vs. 2.68 ± 0.55 *p* < 0.001	2.02 ± 0.77 vs. 2.18 ± 0.56 vs. 2.08 ± 0.45 *p* = 0.228	1.83 ± 0.62 vs. 2.11 ± 0.68 vs. 1.89 ± 0.35 *p* = 0.054	1.67 ± 0.62 vs. 1.87 ± 0.63 vs. 1.77 ± 0.30 *p* = 0.043	2.39 ± 0.63 vs. 2.62 ± 0.53 vs. 2.55 ± 0.40 *p* = 0.082
Working Department [ICU (90) vs. Other (80)]	3.03 ± 0.69 vs. 3.19 ± 0.52 *p* = 0.083	3.62 ± 0.68 vs. 3.54 ± 0.46 *p* = 0.347	2.94 ± 0.89 vs. 2.79 ± 0.57 *p* = 0.168	3.29 ± 0.53 vs. 3.00 ± 0.46 *p* < 0.001	3.48 ± 1.06 vs. 3.10 ± 0.67 *p* = 0.005	2.15 ± 0.79 vs. 1.98 ± 0.54 *p* = 0.105	1.88 ± 0.67 vs. 1.93 ± 0.55 *p* = 0.638	1.81 ± 0.73 vs. 1.64 ± 0.37 *p* = 0.057	2.49 ± 0.70 vs. 2.44 ± 0.44 *p* = 0.578
Working Position [Doctor (40) vs. Nurse (110) vs. Other (20)]	3.28 ± 0.69 vs. 3.02 ± 0.61 vs. 3.20 ± 0.40 *p* = 0.032	3.74 ± 0.57 vs. 3.55 ± 0.61 vs. 3.43 ± 0.36 *p* = 0.107	3.08 ± 0.73 vs. 2.77 ± 0.80 vs. 3.00 ± 0.46 *p* = 0.105	3.30 ± 0.52 vs. 3.14 ± 0.53 vs. 2.92 ± 0.32 *p* = 0.023	3.29 ± 1.00 vs. 3.39 ± 0.92 vs. 2.80 ± 0.52 *p* = 0.028	1.80 ± 0.53 vs. 2.17 ± 0.75 vs. 2.02 ± 0.44 *p* = 0.011	1.69 ± 0.55 vs. 1.84 ± 0.53 vs. 2.64 ± 0.66 *p* < 0.001	1.55 ± 0.40 vs. 1.79 ± 0.68 vs. 1.77 ± 0.26 *p* = 0.078	2.29 ± 0.58 vs. 2.50 ± 0.61 vs. 2.62 ± 0.39 *p* = 0.062
Working experience [0–5 (53) vs. 6–10 (52) vs. 11–15 (31) vs. >15 (34)]	3.09 ± 0.71 vs. 3.26 ± 0.47 vs. 2.93 ± 0.58 vs. 3.07 ± 0.68 *p* = 0.116	3.70 ± 0.60 vs. 3.51 ± 0.42 vs. 3.41 ± 0.65 vs. 3.64 ± 0.69 *p* = 0.113	2.93 ± 0.81 vs. 2.90 ± 0.50 vs. 2.68 ± 1.01 vs. 2.91 ± 0.75 *p* = 0.482	3.26 ± 0.62 vs. 3.01 ± 0.41 vs. 3.02 ± 0.48 vs. 3.33 ± 0.43 *p* = 0.005	3.53 ± 0.93 vs. 2.99 ± 0.68 vs. 3.20 ± 1.07 vs. 3.50 ± 0.95 *p* = 0.010	2.02 ± 0.82 vs. 2.02 ± 0.48 vs. 2.18 ± 0.58 vs. 2.11 ± 0.82 *p* = 0.674	1.88 ± 0.61 vs. 1.89 ± 0.60 vs. 1.95 ± 0.52 vs. 1.90 ± 0.73 *p* = 0.964	1.71 ± 0.67 vs. 1.76 ± 0.46 vs. 1.67 ± 0.43 vs. 1.77 ± 0.77 *p* = 0.901	2.35 ± 0.66 vs. 2.50 ± 0.44 vs. 2.54 ± 0.61 vs. 2.51 ± 0.64 *p* = 0.405
Source of information [Internet (114) vs. Television (37) vs. Other (19)]	3.08 ± 0.64 vs. 3.14 ± 0.49 vs. 3.19 ± 0.71 *p* = 0.696	3.58 ± 0.59 vs. 3.39 ± 0.44 vs. 3.94 ± 0.65 *p* = 0.004	2.85 ± 0.79 vs. 2.78 ± 0.53 vs. 3.16 ± 0.90 *p* = 0.261	3.20 ± 0.52 vs. 2.86 ± 0.42 vs. 3.47 ± 0.44 *p* < 0.001	3.34 ± 0.91 vs. 2.96 ± 0.72 vs. 3.74 ± 1.10 *p* = 0.008	2.08 ± 0.61 vs. 2.20 ± 0.78 vs. 1.73 ± 0.83 *p* = 0.018	1.77 ± 0.50 vs. 2.28 ± 0.65 vs. 1.97 ± 0.76 *p* < 0.001	1.71 ± 0.55 vs. 1.91 ± 0.70 vs. 1.49 ± 0.57 *p* = 0.015	2.41 ± 0.57 vs. 2.75 ± 0.50 vs. 2.22 ± 0.68 *p* = 0.001

COVID-19: Coronavirus Disease 2019; DASS21: Depression, Anxiety, Stress; Fear of COVID-19 Scale: Fear of COVID-19; GHL: General Hospital of Larissa; GHT: General Hospital of Trikala; UHL: University Hospital of Larissa; WHOQOL-BREF: Mental Health, Physical Health, Quality of Life, Work Environment. Comparisons between two groups were made using *t*-test, whereas comparisons between three or more groups were performed using one-way ANOVA.

**Table 5:** Correlations between variables concerning the quality of life, fear of COVID-19 negative emotions and other factors.

Variables	1 Physical Health	2 Mental Health	3 Work Environment	4 Social Relations	5 Quality of Life	6 Fear of COVID-19	7 Stress	8 Anxiety	9 Depression	10 Effect of Mass Media	11 Safety during Work	12 PPEs
1.Physical Health	1											
2.Mental Health	0.543 **	1										
3.Work Environment	0.550 **	0.361 **	1									
4. Social relations	0 554 **	0.333 **	0.618 **	1								
5. Quality of Life	0.377 **	0.311 **	0.229 **	0.145	1							
6. Fear of COVID-19	−0.434 **	−0.453 **	−0.344 **	−0.310 **	−0.218 **	1						
7. Stress	−0.557 **	−0.528 **	−0.442 **	−0.477 **	−0.307 **	0.548 **	1					
8. Anxiety	−0.558 **	−0.436 **	−0.326 **	−0.350 **	−0.226 **	0.593 **	0.649 **	1				
9. Depression	−0.452 **	−0.426 **	−0.458 **	−0.432 **	−0.267 **	0.381 **	0.600 **	0.432 **	1			
10.Effect of mass media	0.198 **	0.005	0.286 **	0.308 **	0.142	−0.082	−0.151 *	−0.224 **	−0.052	1		
11. Safety during work	0.338 **	0.195 *	0.506 **	0.348 **	0.192 *	−0.356 **	−0.230 **	−0.284 **	−0.262 **	0.211 **	1	
12. PPEs	0.244 **	0.312 **	0.241 **	0.055	0.284 **	−0.210 **	−0.217 **	−0.184 *	−0.210 **	−0.085	0.305 **	1

* *p* < 0.05; ** *p* < 0.01.

**Table 6:** Evaluation of possible predictors of quality of life.

	Mental Health	Physical Health	Quality Of Life	Environment	Social Relations	Fear of COVID-19	Depression	Anxiety	Stress
Gender (Male vs. Female)	b = −0.168 *p* = 0.024	-	-	-	-	-	-	-	-
Marital Status (Divorced-Windowed vs. unmarried vs. married	-	-	-	Divorced-windowed (b = −0.178, *p* = 0.007)	-	-	-	-	-
Working Institution (UHL vs. GHL vs. GHT)	-	-	-	-	-	-	-	-	-
Working Department (ICU vs. Other)	-	-	-	-	-	-	-	-	-
Working Position (Doctor vs. Nurse vs. Other)	-	-	-	-	-	-	Other (b = 0.382, *p* < 0.001)	-	-
Working experience 0–5 vs. 6–10 vs. 11–15 vs. >15	-	-	-	-	-	-	-	-	-
Source of information Internet vs. Television vs. Other	-	Other (b = 0.186, *p* = 0.010)	-	Other (b = 0.139, *p* = 0.029)	-	-	Television (b = 0.200, *p* = 0.006)	-	Television (b = 0.215, *p* = 0.004)
Effect of mass media	-	b = 0.146 *p* = 0.046	-	-	b = 0.180 *p* = 0.020	-	-	-	-
Safety during work	-	b = 0.228 *p* = 0.004	-	b = 0.290 *p* < 0.001	b = 0.180 *p* = 0.021	b = −0.301 *p* < 0.001	-	b = −0.169 *p* = 0.048	-
PPEs	b = 0.218 *p* = 0.007	b = 0.170 *p* = 0.029	b = 0.252 *p* = 0.002	b = 0.233 *p* = 0.001	-	-	-	b = −0.175 *p* = 0.033	b = −0.198 *p* = 0.013
Model									
*p*-value	<0.001	<0.001	<0.001	<0.001	<0.001	<0.001	<0.001	0.001	<0.001
AdjR^2^	0.099	0.169	0.086	0.384	0.176	0.172	0.254	0.189	0.129

Only significant variables are reported. GHL: General Hospital of Larissa; GHT: General Hospital of Trikala; ICU: Intensive Care Unit; UHL: University Hospital of Larissa.

## Data Availability

Data will be available upon reasonable request.

## Appendix A

### Appendix A.1. Methods

#### Survey Questionnaire

The distributed questionnaire consisted of 59 questions divided into four sections. The first section referred to the demographic and professional experience data using seven questions. The second section included 10 questions about attitudes concerning the pandemic, the source of information regarding COVID-19, feeling of fear during the first and second pandemic period, attitude towards vaccination (the study period covered the first pandemic wave, before the vaccine was launched, and the second wave during which the first vaccine was available), the impact of the mass media during the COVID-19 period, feeling safe while performing work tasks and the adequacy of the provision of protection measures according to the International Guidelines (Personal Protective Equipment, PPEs) [41]. The question concerning the source of information had three answers internet (referring to the social media), television and other; other covered sources such as scientific resources of information, PubMed, Scopus. The third section included 18 questions on quality of life, according to the World Health Organization Quality of Life Assessment -WHOQOL-BREF questionnaire [14]. The validity of the questionnaire has been proved acceptable for physical (a = 0.668), mental health (a = 0.686) and working environment (a = 0.676) and satisfactory for social relations (a = 0.758) [14]. The questions are answered on a 5-point Likert scale from 1 to 5.

The fourth section measures the negative emotional states of depression, anxiety and stress using the Depression Anxiety and Stress Scale 21 (DASS21) questionnaire [15] and six questions from the Fear of COVID-19 Scale questionnaire [18], which have been validated in previous surveys [19]. The DASS21 questionnaire has indicated acceptable reliability for stress (a = 0.603) and depression (a = 0.650) and satisfactory for anxiety (a = 0.706). The Fear of COVID-19 Scale showed high reliability (a = 0.806).

## Appendix B

### Appendix B.1. Questionnaire

#### Appendix B.1.1. Demographics

1. Gender

☐ Male
☐ Female

2. Age

☐ Up to 30
☐ 31–40
☐ 41–50
☐ >50

3. Marital status

☐ Unmarried
☐ Married
☐ Divorced
☐ Widower

4. Working Institution

☐ University Hospital of Larissa (UHL)
☐ General Hospital of Larissa (GHL)
☐ General Hospital of Trikala (GHT)

5. Department of Work

☐ ICU
☐ Emergency Department
☐ Internal Medicine Department
☐ Respiratory Department
☐ Department of Infectious Diseases
☐ Other

6. Profession

☐ Doctor
☐ Nurse
☐ Physiotherapist
☐ Paramedic
☐ Ward Assistant
☐ Cleaning services

7. Working experience

☐ Up to 5
☐ 6–10
☐ 11–15
☐ >15

#### Appendix B.1.2. Impact of the Pandemic on Daily Life

8. Have you been infected by coronavirus?

☐ Yes
☐ No

9. Has anyone in your close environment been infected by coronavirus? (Multiple answers)

☐ Home environment
☐ Working environment
☐ Friendly Environment
☐ All the above
☐ Nobody

10. During the period of COVID-19, where did you get the information, you might need in your daily life?

☐ Internet
☐ Television
☐ Other

11. Did you feel scared about the COVID-19 pandemic?

☐ Yes
☐ No

If *YES,* when did you feel most afraid?

☐ The first period
☐ The second period

12. As is known, the vaccine against the corona virus disease is now also available in our country. Have you been waiting for the release of the vaccine (before December 2020) to eliminate the COVID-19 pandemic?

☐ Yes
☐ No

13. Have you been vaccinated against the coronavirus disease?
☐ Yes
☐ No

14. As a health professional, would you recommend vaccination against coronavirus disease?

☐ Yes
☐ No

15. How do you rate the effect of the Mass Media during the period of COVID-19?

☐ Very bad
☐ Bad
☐ Moderate
☐ Good
☐ Very good

16. During the COVID-19 period, how safe did you feel during your work duties with hospitalized patients with coronavirus?

☐ Not at all
☐ A bit
☐ A lot
☐ Very much
☐ Absolutely

17. During the COVID-19 period, did your Working Institution provide and continue to provide you with the prescribed protection measures in accordance with International Guidelines?

☐ Not at all
☐ A bit
☐ A lot
☐ Very much
☐ Absolutely

#### Appendix B.1.3. World Health Organization Quality of Life Assessment-WHOQOL-BREF

18. How would you rate your quality of life during the COVID-19 period?

☐ Very bad
☐ Bad
☐ Moderate
☐ Good
☐ Very good

19. Do you feel that, during the period of COVID-19, possibly physicalizing stress, prevented you from doing things you have to do?

☐ Not at all
☐ A bit
☐ Moderate
☐ Much
☐ Extreme

20. Did you need, during the period of COVID-19, any medical treatment (counseling, anxiolytics) to function in your daily life?

☐ Not at all
☐ A bit
☐ Moderate
☐ Much
☐ Extreme

21. How healthy was your natural environment, during the period of COVID-19?

☐ Not at all
☐ A bit
☐ Moderate
☐ Much
☐ Extreme

22. How much were you enjoying life, during the period of COVID-19?

☐ Not at all
☐ A bit
☐ Moderate
☐ Much
☐ Extreme

23. During the period of COVID-19, how well did you concentrate on something you do?

☐ Not at all
☐ A bit
☐ Moderate
☐ Much
☐ Extreme

24. During the period of COVID-19, how safe did you feel in your daily life?

☐ Not at all
☐ A bit
☐ Moderate
☐ Much
☐ Extreme

25. During the period of COVID-19, did you have the necessary energy for the activities of daily life?

☐ Not at all
☐ To small degree
☐ Moderate
☐ To high degree
☐ Absolutely

26. During the period of COVID-19, did you have the necessary money to meet your needs?

☐ Not at all
☐ To small degree
☐ Moderate
☐ To high degree
☐ Absolutely

27. During the period of COVID-19, were you able to do any activities in your free time?

☐ Not at all
☐ To small degree
☐ Moderate
☐ To high degree
☐ Absolutely

28. How satisfied were you with your sleep, during the period of COVID-19?

☐ Very dissatisfied
☐ Moderate dissatisfied
☐ Nor satisfied neither dissatisfied
☐ Moderate satisfied
☐ Very satisfied

29. During the period of COVID-19, how satisfied were you with your ability to work?

☐ Very dissatisfied
☐ Moderate dissatisfied
☐ Nor satisfied neither dissatisfied
☐ Moderate satisfied
☐ Very satisfied

30. During the period of COVID-19, how satisfied were you with your personal relationships?

☐ Very dissatisfied
☐ Moderate dissatisfied
☐ Nor satisfied neither dissatisfied
☐ Moderate satisfied
☐ Very satisfied

31. During the period of COVID-19, how satisfied were you with your sex life?

☐ Very dissatisfied
☐ Moderate dissatisfied
☐ Nor satisfied neither dissatisfied
☐ More satisfied
☐ Very satisfied

32. During the period of COVID-19, how satisfied were you with the support you received from your friends?

☐ Very dissatisfied
☐ Moderate dissatisfied
☐ Nor satisfied neither dissatisfied
☐ Moderate satisfied
☐ Very satisfied

33. During the period of COVID-19, how satisfied were you with your work environment (Colleagues, Supervisor, Manager)?

☐ Very dissatisfied
☐ Moderate dissatisfied
☐ Nor satisfied neither dissatisfied
☐ Moderate satisfied
☐ Very satisfied

34. During the period of COVID-19, how satisfied were you with your access to various health services?

☐ Very dissatisfied
☐ Moderate dissatisfied
☐ Nor satisfied neither dissatisfied
☐ Moderate satisfied
☐ Very satisfied

35. During the period of COVID-19, how often did you have negative feelings, such as gloomy mood, hopelessness, anxiety, depression?

☐ Never
☐ Seldom
☐ Sometimes
☐ Often
☐ Always

#### Appendix B.1.4. Fear of COVID-19 Scale and Dass 21

**Fear of COVID-19**	**Not at All**	**A Bit**	**Much**	**Very Much**	**Extreme**
**Questions**	**1**	**2**	**3**	**4**	**5**
36. I am very scared about the coronavirus					
37. When I think about the coronavirus, I feel insecure					
38. My hands sweat when I think about the coronavirus.					
39. I am afraid of losing my life because of the coronavirus.					
40. When I see news about the coronavirus on social media, I get nervous and anxious.					
41. I cannot sleep because I am worried about contracting the coronavirus.					
Stress-Anxiety-Depression during the period of COVID-19 in the workplace (DASS 21)	1	2	3	4	5
42. I could not calm myself down					
43. I felt my mouth dry?					
44. I could not feel any positive emotion.					
45. I had difficulty in breathing (breathing too fast, shortness of breath without physical exertion)?					
46. I find it difficult to take initiative about certain things?					
47. I exaggerated in my reactions to various situations I faced?					
48. My hands were shaking					
49. I was often nervous					
50. I was worried about situations where I might panic and look foolish to others.					
51. I felt that I had nothing interesting to anticipate					
52. I found myself feeling down					
53. I found it hard to relax					
54. I felt deressed and dissapointed					
55. I could not tolerate anything that kept me from continuing what I was doing?					
56. I came close to feeling panic?					
